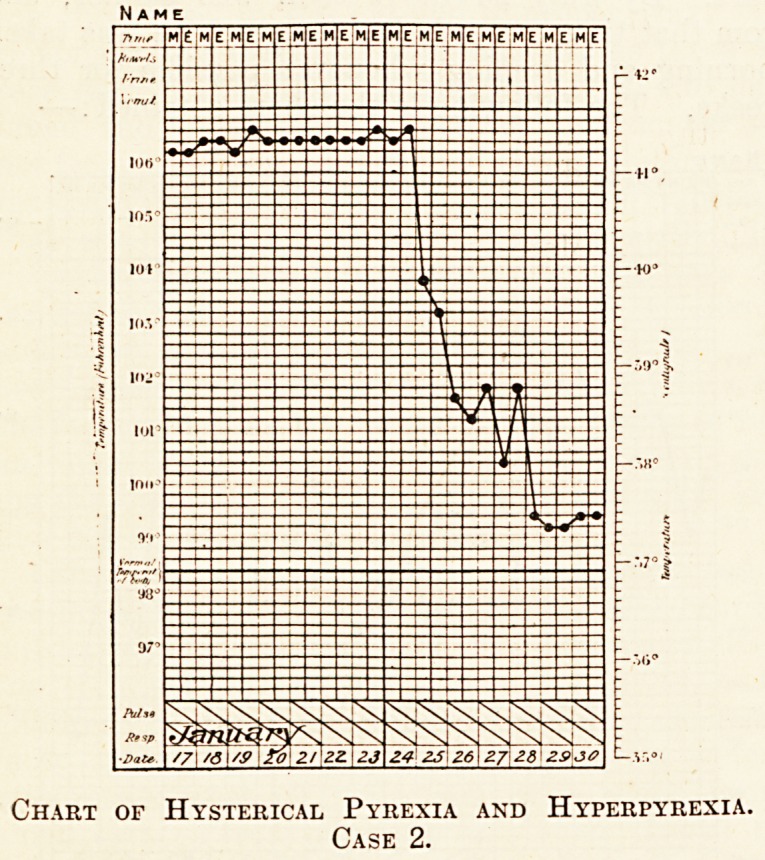# Functional or Hysterical Pyrexia

**Published:** 1908-05-16

**Authors:** 


### Clinical Points.

### FUNCTIONAL OR HYSTERICAL PYREXIA.

Pyrexia is very nearly always a symptom of importance. Nevertheless in certain patients, especially those of hysterical or neurotic temperament, the thermometer may register temperatures to which no particular importance can be attached, except in so far as they may cause difficulties in diagnosis. When the pyrexia is slight, it is next to impossible to be sure whether it is due to functional or organic trouble, but when it is extreme there is much less difficulty in interpreting it; for in the functional cases there is no symptom of visceral derangement at all proportional to the pyrexia, nor is there any clinical change when the pyrexia abates or disappears. It would seem that the thermometer, for some reason, registers a temperature which is not that of the circulating blood, for the viscera show no signs of being bathed in superheated fluid. Nevertheless, the pyrexia is not artificial in the sense that the thermometer has been deliberately interfered with by the patient. There are malingerers, of course, who know how to make the mercury rise abnormally high, but apart from these there are also patients in whom the temperature, registered in the ordinary way with all precautions to avoid fraud, is abnormally high without there being any organic disease.

The following are two cases in point:—

The first is that of a young unmarried woman who had for years suffered from a variety of the symptoms of hysteria major. She was subject to convulsive paroxysms, in which she was extremely violent and hysterical. She had also suffered from transitory paralyses; profound sensory disorders, both of cutaneous sensibility and of the special senses; and so on. One morning, after a very violent hysterical and convulsive seizure, she was attacked with complete paralysis and anæsthesia of the whole of the left side, with the exception of the face. She was conveyed to a hospital, and there she had more than thirty convulsive seizures in twelve days. She remained thus for several weeks, sometimes passing two or three days at a time without eating, without micturating, and in a state of absolute mutism. Then she seemed to wake up as though from a dream, and for several days appetite and speech would return, urine would be voided, and after an interval a fresh seizure, with its subsequent symptoms, would occur. At first all sorts of treatment were tried. Later, simple nursing alone was resorted to. One morning, after one of the most violent attacks of wild hysteria, the skin seemed to be hot and dry, and the pulse frequent. The axillary temperature was taken, and it was found to be 102.2° F., the pulse rate being 98 at the same time. The most careful examination failed to discover any visceral trouble, but it was thought that something might have developed by the morrow. This was not so, however, and throughout the period of observation there was not the slightest sign of any organic visceral mischief. The temperature was 101.4° F. on the second day. Then followed two days during which it was impossible to use the thermometer upon the patient, who was a prey to a series of most violent attacks of hysterical convulsions which ended either in an interminable hiccough or else in sobs and floods of tears. By July 23 the woman was calmer, and from that time onwards her temperature was taken morning and evening whenever possible for three weeks. The following is the chart obtained:—

CHART OF HYSTERICAL PYREXIA AND HYPERPYREXIA. CASE 1.

It will be seen that for the whole period of three weeks there was continuous pyrexia. Notwithstanding that the nurse never left the patient whilst the temperature was being taken, the precaution of taking it oneself and independently both in the axilla and in the rectum simultaneously was adopted, different thermometers being used at different times. The rectal temperatures were sometimes higher, sometimes lower, than those obtained in the axilla, but the difference was never great. It is clear that there was no malingering on the patient's part.

Notwithstanding persistence of the pyrexia for these three weeks, the patient's respirations remained natural except during the paroxysms of hysteria; the alimentary system was not affected in the least, although it is always the first to be upset in cases of real fever; the tongue remained moist, and when the patient passed days without eating this was not because of gastric disorder, but the result of nervous disturbance.

After three weeks there was a sort of crisis of defervescence, like that of lobar pneumonia; but unlike what occurs in the latter disease, the general condition showed no sudden alteration at the same time; it remained just what it had been before, and it was neither better nor worse than it had been during the pyrexial interval.

At different times there had been a question as to whether the patient might not be really an epileptic, but the attacks from which she suffered were typically hysterical; there was no initial cry, consciousness was never completely lost; the convulsions were entirely clonic, without a tonic stage; the tongue was never bitten, and there was never a true coma at the end of an attack, the latter nearly always terminating either in deep sobbings or else

Chart of Hysterical Pyrexia and Hyperpyrexia. Case 2.

in long-continued and frequent hiccough. Moreover, hyperpyrexia in an epileptic is always a very grave matter, whereas in this patient there was never any evidence of danger. In fine, the case seemed to be one of unusually severe hysteria, accompanied at one period by extreme hysterical pyrexia.

The other case was less severe as regards the general symptoms of hysteria, but even more marked than the above in respect of pyrexia and periods of hyperpyrexia. She was a woman of very nervous temperament, and almost every evening about seven o'clock she was seized by an "attack of nerves," which usually lasted without intermission until about one in the morning; in these attacks she was extremely restless and complained of all sorts of subjective sensations, of which the chief were feelings of oppressive heat and ideas that her limbs were being bent and strained to the breaking-points of the bones.

For more than a year past she had been noted to have bouts of pyrexia, considerable in degree, coming and going suddenly without any apparent cause other than her neuroses. At the beginning of one November the patient had a continuous morning and evening temperature of 103° F., rising during December to 104° F., and reaching 105.8° F. on December 24. The axillary temperatures taken during the latter half of January are given in the second chart.

The pyrexia lasted continuously for three months, and it was never accompanied by any visceral trouble; even appetite was not interfered with. When the trouble ceased, it did so instantaneously, without any convalescence. Loss of weight and strength were not in evidence, as they are in any ordinary febrile illness, and this notwithstanding the fact that there had been hyperpyrexia repeatedly. It seems almost certain that the hyperpyrexia in these cases is altogether of a different kind to that seen in such illnesses as lobar pneumonia, for example. Its main importance lies in the fact that, if correctly diagnosed, it is free from danger.

## Figures and Tables

**Figure f1:**
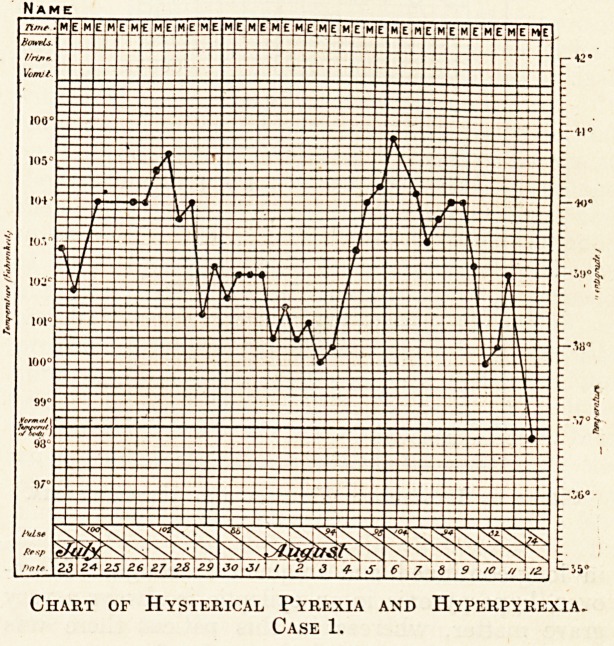


**Figure f2:**